# Transfer from goal-directed behavior to stimulus-response habits and its modulation by acute stress in individuals with risky gaming behavior

**DOI:** 10.1038/s41598-024-73899-3

**Published:** 2024-10-29

**Authors:** Anna M. Schmid, Tobias A. Thomas, Stefan Blümel, Nicolas K. Erdal, Silke M. Müller, Christian J. Merz, Oliver T. Wolf, Matthias Brand, Astrid Müller, Sabine Steins-Loeber

**Affiliations:** 1 Department of Clinical Psychology and Psychotherapy, Otto-Friedrich-University of Bamberg, Bamberg, Germany; 2 Department of Psychosomatic Medicine and Psychotherapy, Hannover Medical School, Hannover, Germany; 3 General Psychology: Cognition, Faculty of Computer Science, University of Duisburg-Essen, Duisburg, Germany; 4 Center for Behavioral Addiction Research (CeBAR), Center for Translational Neuro- and Behavioral Sciences, University Hospital Essen, University of Duisburg-Essen, Essen, Germany; 5 Erwin L. Hahn Institute for Magnetic Resonance Imaging, Essen, Germany; 6 Department of Cognitive Psychology, Institute of Cognitive Neuroscience, Faculty of Psychology, Ruhr University Bochum, Bochum, Germany

**Keywords:** Gaming disorder, Pavlovian-to-instrumental transfer, Habitual behavior, Conditioning, Cues, Stress, Human behaviour, Addiction

## Abstract

Habitual responses towards addiction-related cues play a relevant role in the development and maintenance of addictions. Such automatic responses may be more likely under stress, as stress has been shown to induce a shift from goal-directed to habitual behavior. The current study investigated these mechanisms in risky gaming behavior. Individuals with risky gaming behavior (*n* = 68), as established by a structured clinical interview, and a matched control group (*n* = 67) completed a Pavlovian-to-Instrumental Transfer (PIT) paradigm with gaming-related cues and rewards. After the Pavlovian training, participants underwent a stress (Trier Social Stress Test) or control condition before performing the instrumental training and the transfer phase of the PIT paradigm. To assess habitual behavior, the gaming-related rewards were devalued after half of the transfer phase. In both groups, gaming-related cues enhanced the choice of the gaming-related reward and this gaming PIT effect was reduced, however, not eliminated by the devaluation. Unexpectedly, stress did not significantly increase responding for the gaming-related reward in participants aware of the stimulus-outcome associations, however seemed to enhance habitual responding in unaware participants. Our findings underline the relevance of gaming-related cues in triggering habitual responses, which may undermine attempts to change a problematic gaming behavior.

**Supplementary Information:**

The online version contains supplementary material available at 10.1038/s41598-024-73899-3.

## Introduction

Individuals often continue to engage in addictive behaviors even when the negative consequences largely outweigh the positive short-term rewards and even if they have the best intentions not to do so. This observation is often explained with a shift from goal-directed behavior to stimulus-response habits in the course of addiction^1,2^, although the nature of habits in addictions is still debated^3,4^. As a consequence of the potential involvement of habits in addictions, the addictive behavior may increasingly manifest itself in automatic and inflexible responses towards conditioned addiction-related cues^1,2^, with cognitive control over the behavior becoming more and more difficult^1,2^. An involvement of stimulus-response habits, or at least seemingly habitual or compulsive behaviors, is assumed not only for substance-use disorders^1^, but also for behavioral addictions^5,6^, a category that has been newly added to the eleventh revision of the International Classification of Diseases (ICD-11)^7^ and, besides gambling disorder, lists gaming disorder as a disorder due to addictive behaviors.

From the perspective of learning theory, the potential transition from goal-directed to habitual behavior could evolve from the interaction of instrumental and Pavlovian learning mechanisms^1,8,9^. A paradigm that allows to study the interaction of these two learning mechanisms is the Pavlovian-to-Instrumental Transfer (PIT) paradigm^10,11^. This paradigm independently establishes stimulus-outcome (S-O) associations during a Pavlovian training phase and response-outcome (R-O) associations during an instrumental training phase, which are then tested together in the transfer phase, with the aim to assess how conditioned cues affect instrumental responding^10,11^. A typical observation in studies using the PIT paradigm is that conditioned stimuli associated with a certain outcome can trigger instrumental responses to earn this outcome, a mechanism that is called specific PIT effect^11,12^.

Much of the research on PIT effects in addiction has focused on substance use disorders ^e.g., 13–19^. A consistent finding among these studies is that cues associated with tobacco or alcohol can increase instrumental responding for tobacco- or alcohol-related outcomes^13–19^. Please note that some of these PIT paradigms omitted the Pavlovian training phase and instead relied on naturally conditioned cues, like pictures of alcoholic drinks or cigarettes^13–15,18^. In studies that included a Pavlovian training phase, a PIT effect was only observed for individuals who had acquired awareness of the stimulus-outcome associations during Pavlovian training^16,17^. In contrast to substance use disorders, research regarding the PIT effect in behavioral addictions is still scarce. To the best of our knowledge, only three studies used the PIT paradigm in the context of problematic gaming behavior (used here as umbrella term for both subclinical and clinical levels of gaming problems) or pathological gaming behavior (defined here as meeting the criteria of gaming disorder). Piloting a PIT paradigm with gaming-related rewards and cues in a convenience sample, Vogel et al.^20^ were able to demonstrate a gaming-related PIT effect. In addition, the severity of problematic gaming behavior predicted the strength of expectancies regarding the stimulus-outcome associations, which in turn was associated with an increased gaming PIT effect^20^. A second study, applying a similar version of the PIT paradigm, compared individuals with gaming disorder to a control group and found a greater gaming-related PIT effect in individuals with gaming disorder compared to control participants^22^. Finally, Xu et al.^23^ also studied PIT effects in individuals with gaming disorder, however used a more complex PIT paradigm, which required participants to perform (go trials) or withhold responses (no-go trials) to gain monetary rewards. The effect of monetary cues on instrumental behavior was larger in individuals with gaming disorder compared to control participants, however, surprisingly, this did not apply to gaming-related cues.

However, none of these studies examined whether the observed gaming PIT effect was insensitive towards a change in outcome value, hence presenting habitual behavior, which, as outlined above, may be a feature of addictions. Sensitivity towards a change in outcome value is often tested with so-called devaluation procedures, in which the value of one of the outcomes is decreased, for example, by consumption to satiety or taste aversion^24,25^. To test the impact of devaluation on the PIT effect, the outcome devaluation is typically scheduled before the transfer phase^11^. While there is increasing evidence that the PIT effect is sensitive towards outcome devaluation and hence goal-directed^11^, it has not been resolved yet whether this finding also applies to individuals suffering from addiction. The few studies, which investigated the link between addiction and the devaluation sensitivity of addiction-related PIT effects, were conducted in the context of smoking and failed to find an association with dependency or severity of smoking^26,27^.

While studies investigating the devaluation sensitivity of PIT effects in gaming disorder have not been conducted yet, Xu et al.^23^ assessed the effect of outcome devaluation on instrumental responding for monetary rewards. Individuals with gaming disorder and control participants equally reduced responding for the devalued outcome, which implied goal-directed control in both groups. However, a different picture may emerge if analyzing responding in the presence of gaming-related cues.

Stress has been repeatedly related to the development, maintenance, and relapse in substance use disorders ^e.g., 28–30^ and is also considered important in the context of behavioral addictions like gaming disorder^5,31^. On a physiological level, stress activates the sympathetic nervous system and the hypothalamic–pituitary–adrenal (HPA) axis^32^. The activation of the sympathetic nervous system leads to the release of adrenaline and noradrenaline from the adrenal medulla and is responsible for the rapid response to stress, consisting of increases in heart rate, blood pressure, and breathing frequency^33^. The slower acting HPA axis triggers the release of glucocorticoids (mainly cortisol in humans) in the adrenal cortex, which – by binding to glucocorticoid and mineralocorticoid receptors – exert multiple effects in the brain as well as the peripheral nervous system^32,34^. A way how stress may contribute to addiction is by promoting a shift from goal-directed behaviors to stimulus-response habits^25^ – possibly mediated by cortisol^35^ – and by increasing the salience of addiction-related stimuli^36^. Hence, the tendency to respond habitually towards addiction-related cues, which is assumed to develop in the course of addiction, may be especially pronounced under stress.

While there are some studies that investigated the impact of stress on the PIT effect, none of these studies examined how these effects are linked to addiction. Moreover, a clear picture regarding the association between stress and the PIT effect did not emerge from these studies. While Quail et al.^37^ failed to find an impact of self-reported stress and anxiety on specific PIT effects, two studies that experimentally induced stress^16,38^ revealed mixed results. An effect of stress on the PIT effect was observed only by Pool et al.^38^ but not by Steins-Loeber et al.^16^. Yet, both studies relied on the Socially Evaluated Cold Pressor Test to induce stress, and a clearer picture of stress may emerge when using a stress induction which triggers more pronounced cortisol responses like the Trier Social Stress Test (TSST)^39^. There is only one study to date that has combined stress induction with outcome devaluation in a PIT paradigm^40^, hence allowing to test rigorously whether stress increases the likelihood for responding habitually. A significant influence of stress was not observed in this study. However, the stress induction used (appraisal of negative pictures) was rather a mood than a stress manipulation.

Whether individuals engage in the addictive behavior when being stressed may also depend on their level of impulsivity and inhibitory control. Not only are these constructs generally considered relevant in the development and maintenance of addictions ^e.g., 5,41–44^, but there is also initial evidence for a moderating influence of impulsivity on stress effects. In a correlational study by Fox et al.^45^, stress was associated with problematic drinking only in individuals high on impulsivity. Similarly, the interaction of impulsivity and life stress proved to be a significant predictor for problematic gambling, with impulsivity having a stronger influence in the presence of high stress^46^. In light of these findings, impulsivity and the related construct of inhibitory control may also moderate the influence of stress on the PIT effect, however studies examining this interaction are missing.

The current study aimed to investigate habitual responding under stress in individuals with risky gaming behavior compared to matched control participants, using a PIT paradigm in combination with an outcome devaluation procedure. Individuals with risky gaming behavior are individuals who display some symptoms of gaming disorder, without having developed the full picture of gaming disorder. With focusing on risky gaming behavior, we investigated a group that has received little attention so far, as research has mainly focused on individuals with pathological gaming behavior.

We hypothesized that individuals with risky gaming behavior would display an increased responding for addiction-related rewards and a greater gaming PIT effect compared to a control group with non-problematic gaming. In individuals with risky gaming behavior, we expected that the stress response is linked to both an increase in general responding for addiction-related rewards and an enhanced PIT effect. Individual aspects, i.e., trait impulsivity and reductions in stimulus-specific inhibitory control, were expected to enhance this effect. Assuming a shift from goal-directed to habitual behavior in addictions, which may be more pronounced under stress, the effect of the outcome devaluation was hypothesized to be smaller in individuals with risky gaming behavior compared to the control group and to be further reduced under stress.

## Method

### Procedure

The procedure applied and test battery assessed in the present study is part of a multi-center DFG-funded addiction research unit (FOR2974) on affective and cognitive mechanisms of specific Internet-use disorders (ACSID)^47^. The study was conducted from October 2021 to July 2023 and testing took place at the University of Bamberg and the Hannover Medical School. The study was approved by the ethics committee of the University Bamberg, Germany (2019–12/33; 18.12.2019) and the ethics committee of the Hannover Medical School, Germany (9025_BO_K_2020; 17.04.2020) and conducted in accordance with the Declaration of Helsinki. Participant data was pseudonymized using ALIIAS: Anonymization/Pseudonymization with LimeSurvey Integration and two-factor Authentication for Scientific research^48^.

**Fig. 1:**
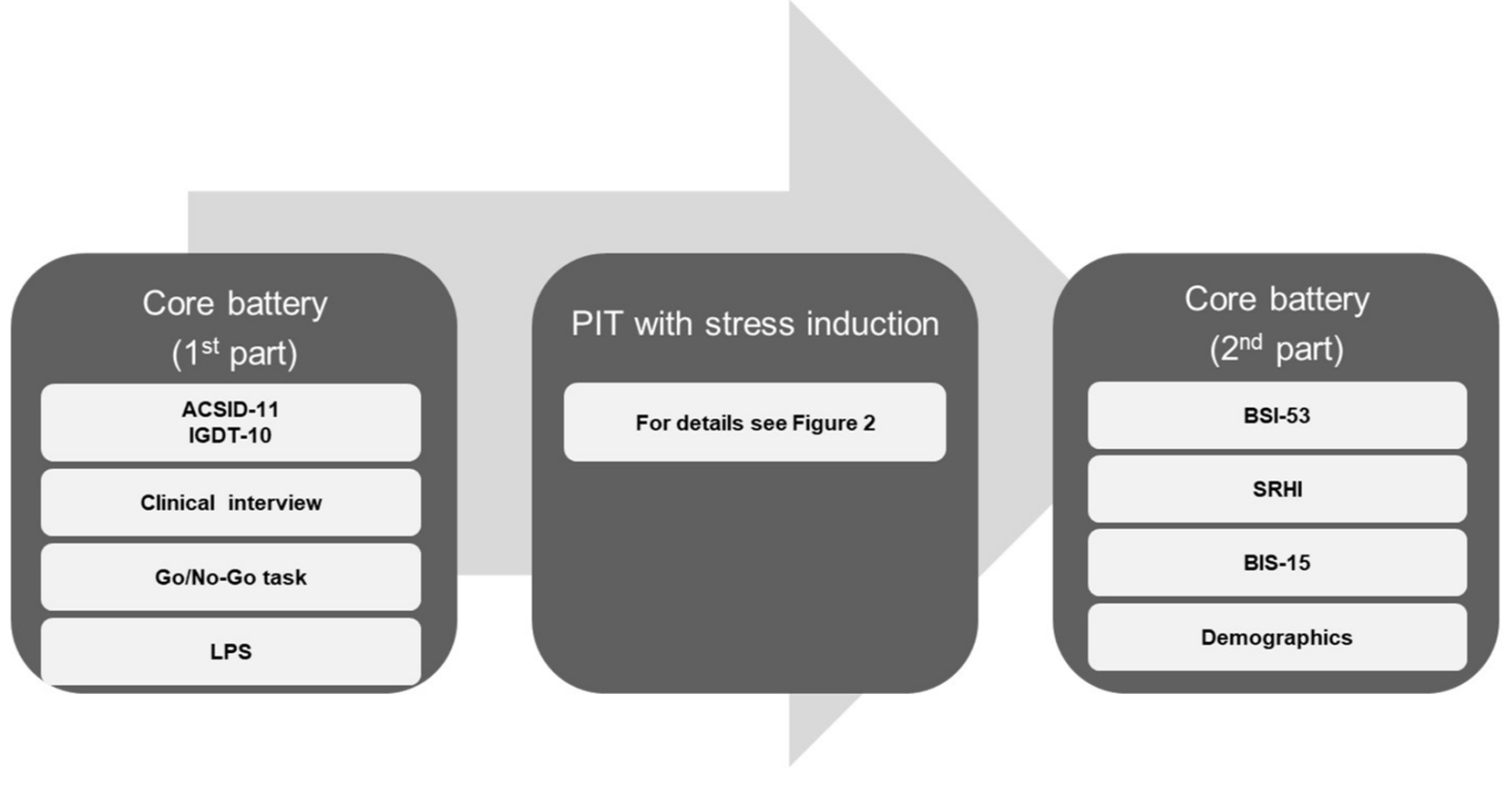
Study procedure. The figure gives an overview of the study procedure. The core battery consisted of a comprehensive set of questionnaires and tasks, however, in the figure, only instruments relevant for the current study are displayed. *ACSID-11* Assessment of Criteria for Specific Internet-use Disorders, *IGDT-10* Ten-Item Internet Gaming Disorder Test, *LPS* Leistungsprüfsystem [Performance assessment system], *BSI-53* Brief Symptom Inventory, *SRHI* Self-Report Habit Index, *BIS-15* Barratt Impulsiveness Scale – short version.

After providing informed consent, participants completed the first part of the FOR2974 core battery^47^, a comprehensive battery of questionnaires and paradigms, which were – with exception of the Go/No-Go Task, a logical reasoning task, some questionnaires and the clinical interview – not relevant for the current study but to the overall goal of the research unit. The completion of the first part of this core battery took around four hours. The next part of the laboratory session involved the PIT task and the stress induction, which was scheduled after the Pavlovian training of the PIT paradigm. By performing the stress/control task in the afternoon, we aimed to minimize the effects of diurnal and meal-related fluctuations in cortisol^49^. Following the stress/control task, participants answered some questions and questionnaires (lasting around 5–10 min), before they completed the remaining parts of the PIT paradigm. Finally, participants were debriefed about the stress/control task and performed the second part of the core battery. For their efforts, participants were reimbursed with 10 euros per hour or – if they were psychology students – could receive course credits for their participation. An overview of the study procedure is depicted in Fig. 1.

### Participants

Participants were part of the FOR2974 cohort and comprised 68 individuals with risky gaming behavior as well as 67 control participants. Risky gaming behavior was operationalized as meeting at least two, but not more than four of the nine DSM-5 criteria for gaming disorder^50^. The control group was matched regarding age and gender and consisted of individuals who reported gaming at least occasionally, but did not fulfil more than one DSM-5 criterion. We aimed to match participants on the individual level, by matching each participant with risky gaming behavior with a control participant of the same gender and in the same 5-year age interval. In some cases, when we did not find a suitable control participant, we deviated from this approach, but made sure that it did not lead to significant age or gender differences between the groups.

Participants were recruited from the University of Bamberg, the Hannover Medical School as well as from the general population by posts on social networks, mailing lists, flyers, and word-of-mouth recommendations. Individuals interested in the study were invited to a 30-minute telephone screening to assess inclusion as well as exclusion criteria and to screen for symptoms of gaming disorder. The final group allocation (at risk versus control) was undertaken at the testing day and was based on a structured clinical interview.

Inclusion criteria for all participants were age between 18 and 65 years and high proficiency in German. Main exclusion criteria were learning or developmental disorders, acute suicidal ideations, psychosis, substance-use disorder (except tobacco), frequent consumption of any psychoactive substances known to interfere with the performance in cognitive tasks, use of medication known to influence the HPA axis and a BMI outside the range of 17–31 kg/m^2^. Women were not tested during their menstruation phase, since a substantial percentage of women experience discomfort during this cycle phase. Finally, we excluded participants who displayed problematic patterns of online shopping to avoid confounding effects due to the shopping-related pictures in our PIT paradigm (see description below).

### Measures

#### Pavlovian-to-instrumental transfer (PIT) paradigm

We used a PIT paradigm with gaming- and shopping-related rewards and cues as developed in our previous research (see Vogel et al.^20^), however, we added a devaluation procedure. Additionally, we used eye-tracking during the Pavlovian training phase to obtain a more objective indicator for conditioning. Hence, when performing the PIT paradigm, participants were seated in front of the computer with their chin resting in the desktop-mount of the eye-tracker.

**Fig. 2:**
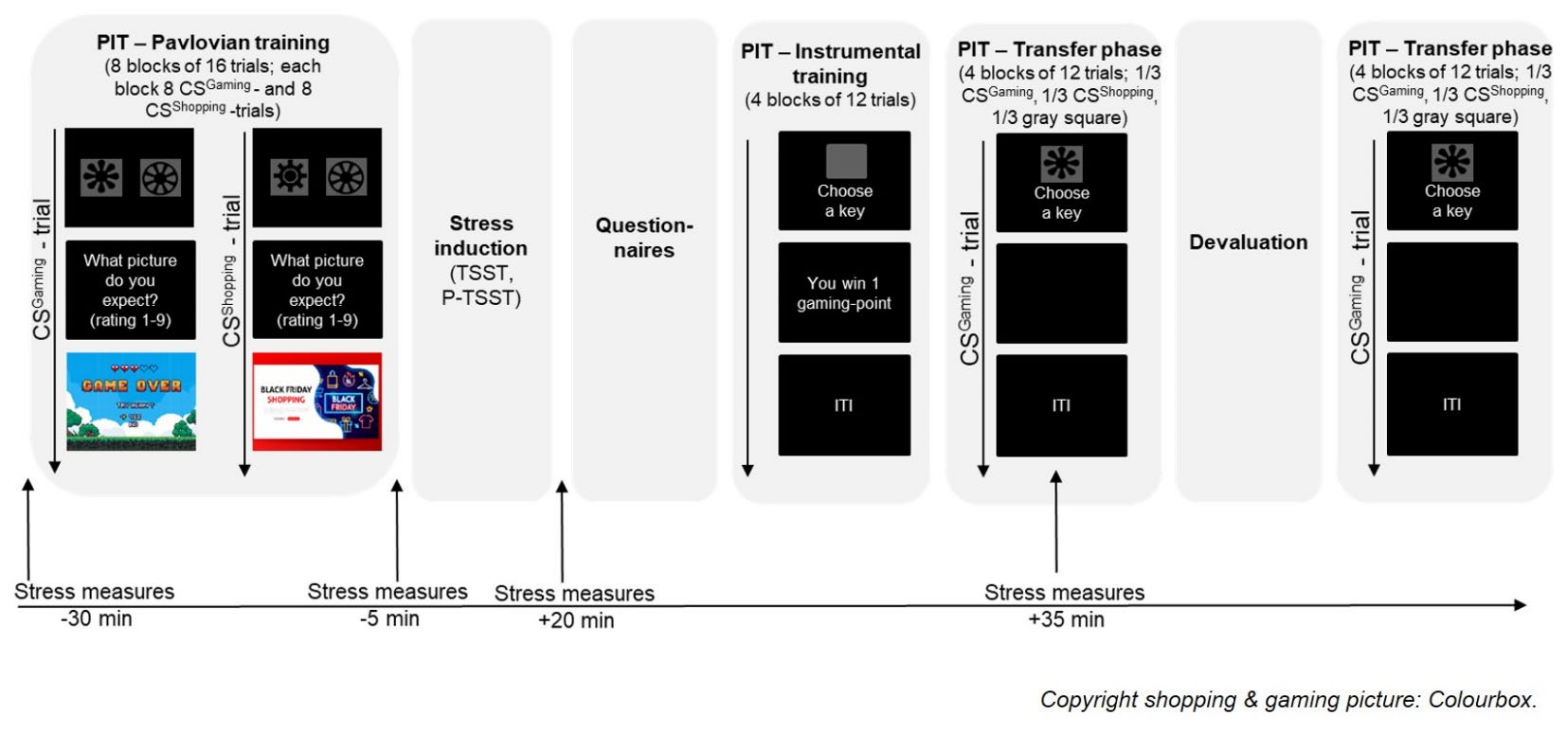
PIT paradigm with stress induction and devaluation. The figure gives an overview of the different phases of the PIT paradigm and the timing of the stress induction and the corresponding stress measurements. The time points of the stress measures refer to stress onset. The shopping and gaming pictures displayed are just sample pictures as the original cues used could not be reproduced due to copyright restrictions. The questionnaires scheduled between the Pavlovian and instrumental training involved questions on conditions affecting the cortisol levels (weight, night shifts etc.) and questionnaires not used for the analyses presented here. *PIT* Pavlovian-to-Instrumental-Transfer, *CS* conditioned stimulus, *TSST* Trier Social Stress Test, *P-TSST* placebo version of the Trier Social Stress Test, *ITI* intertrial interval.

The task started with the Pavlovian training phase (see Fig. 2), in which participants had to learn that one abstract stimulus predicted gaming-related pictures and another abstract stimulus predicted shopping-related pictures. Participants were instructed that they would see pictures and would be asked to provide a rating whether a gaming- or shopping-related picture was more likely to follow. The instruction did neither indicate that there was an association between the abstract stimuli and the gaming- or shopping-related pictures nor were participants instructed to respond as fast or accurately as possible. In every trial, either the stimulus predictive for gaming (conditioned stimulus for gaming [CS^Gaming^]) or the stimulus predictive for shopping (conditioned stimulus for shopping [CS^Shopping^]) was presented in combination with one of two control stimuli. The stimulus presentation lasted 2.3 s and subsequently participants were asked if they expected to see a gaming- or shopping-related picture afterwards. Expectancy was rated on a 9-point scale, labelled with 1 = *shopping-picture*, 5 = *I don’t know*, 9 = *gaming-picture*, with the assignment of the shopping-/gaming-label being counterbalanced across the sample. The question and the rating scale were presented until participants provided a rating. After participants had entered their response, one of 64 shopping- or 64 gaming-related pictures (unconditioned stimuli) was displayed for 2.3 s. The pictures used consisted of screenshots from games and shopping sites and had been validated in two pilot studies regarding craving, arousal, valence, and representativeness. To ensure that a greater proportion of participants learnt the experimental contingencies, we used eight blocks of training instead of four blocks as in the original version^20^. The eye tracking procedure made it necessary for the investigator to observe the eye movements of the participants to ensure precise fixation of the fixation cross at the beginning of each trial. However, the investigator did not monitor the expectancy ratings made by the participants. After the stress induction and before the instrumental training, four questions assessed participants’ knowledge regarding the experimental contingencies. For this purpose, the CS^Gaming^ and the CS^Shopping^ were each presented twice, in a random order, and participants had to decide whether a gaming- or shopping-related picture had followed this stimulus in the previous blocks. Detailed results concerning the Pavlovian training phase of our study including the eye-tracking data is published elsewhere^21^.

During instrumental training, participants learnt two instrumental responses (i.e., pressing the “S” or the “G” key), which earned them shopping or gaming points, respectively. Participants were falsely told that they could exchange these shopping and gaming points for shopping- and gaming-related vouchers at the end of the study. Participants were debriefed about the fictitious nature of the gaming and shopping voucher upon completion of the PIT paradigm. Each trial of the instrumental training phase started with the presentation of a gray square. The gray square was displayed for 2.3 seconds and followed by the prompt *“Please choose a key! Press ‘S’ if you want to win shopping points or ‘G’ if you want to win gaming points.”* Participants could win a gaming or shopping point if they pressed at least once during the 2-second response window, however only 50% of the trials were rewarded. When the response time had passed, participants received an immediate feedback, stating *‘You win one gaming point.’*, *‘You win one shopping point.’* or *‘You win nothing.’* The instrumental training consisted of four blocks of 12 trials.

Finally, the transfer phase assessed instrumental responding in the presence of the CS^Gaming^, the CS^Shopping^, and a neutral stimulus (i.e., gray square). Each stimulus was presented in 1/3 of the trials, and the order in which the stimuli were presented throughout the block was randomized. Again, the stimuli were presented for 2.3 s. After stimulus presentation, the prompt to press the “G” or “S” key appeared, followed by a 2-second response window. To prevent further learning^51^, the transfer phase was conducted in extinction. Hence, participants did not receive immediate feedback about their winnings as in the instrumental training, but were only informed about the number of gaming and shopping points earned after the first four blocks (before the devaluation) and at the end of the transfer phase. As in the instrumental training, only 50% of the trials were rewarded and reinforcement was independent of the stimulus shown. The transfer phase consisted of eight blocks with 12 trials. In contrast to Vogel et al.^20^, the gaming-related rewards were devalued after the first half of the transfer phase by telling participants that the amount of gaming points they had won so far already accounted for the maximum value of the gaming voucher. They were informed that they could still win gaming points in the following blocks, but these wins would not translate into a higher value of the gaming voucher. To ensure that only the gaming-related but not the shopping-related rewards were devalued, participants were informed that they were still able to increase the value of the shopping voucher by winning shopping points.

The following outcome measures were computed for the PIT paradigm: Participants were coded as aware of the experimental contingencies in the Pavlovian training if they (1) displayed significantly higher expectancy ratings of gaming pictures in CS^Gaming^ trials compared to CS^Shopping^ trials in the final block of the Pavlovian training^52^ and (2) answered all four questions regarding the experimental contingencies correctly. Furthermore, we calculated two indicators of instrumental responding, the response choice of the gaming-related reward and the magnitude of the gaming PIT effect. Response choice of the gaming-related reward referred to the percentage of trials in which the gaming-related response was chosen compared to all valid trials (i.e., trials in which either the gaming-related or the shopping-related response was performed). The response choice was calculated for each block of the instrumental training and separately for the transfer phase blocks before and after devaluation. The magnitude of the gaming PIT effect was computed as difference between response choice of the gaming-related reward in gaming trials and response choice of the gaming-related reward in trials, in which the neutral stimulus was presented. Analogous to the response choice, the magnitude of the gaming PIT effect was calculated separately for the transfer phase blocks before and after devaluation.

#### Stress induction

Participants were randomly allocated to the Trier Social Stress Test (TSST)^53^, a widely used psychosocial stress test, which has been demonstrated to increase physiological functions (e.g. heart rate) and trigger cortisol secretion^54,55^, or a control condition. The TSST protocol was the following: Participants were led to another room, in which a female and male research assistant, dressed in white lab-coats and sitting behind a table, awaited them. The TSST was framed as fictitious job interview and participants were informed that their task was to deliver a free speech describing their personal job-relevant traits and to perform an arithmetic task afterwards. Furthermore, participants were made to believe that their performance was videotaped and recorded to later analyze their body language and tone of speech. After giving the instructions, the investigator left the room and the 5-min preparation time started. Subsequently, participants gave their speech (5 min) and performed the arithmetic task (5 min). During the performance of the participants, the committee acted in a reserved and distant manner.

As control condition the Placebo-TSST^56^ was used. In this condition, participants had to also deliver a free speech and perform an arithmetic task, but the tasks were easier (e.g., the speech was about a leisure-time related topic) and the stress-evoking elements (camera, committee) were missing. Like the TSST, the Placebo-TSST lasted 15 min.

When allocating individuals towards the two conditions, attention was paid to individuals with risky gaming behavior and control participants being equally distributed between conditions. The final composition of the conditions was as follows: Placebo: 34 individuals with risky gaming behavior and 34 control participants; TSST: 34 individuals with risky gaming behavior and 33 control participants.

#### Stress measurement

Subjective stress and salivary cortisol were measured at four different time points (see Fig. 2): at the start of the PIT paradigm (T1), directly before (T2) and after (T3) the TSST/Placebo-TSST, and after the first two blocks of the transfer phase of the PIT paradigm (T4). We scheduled the last stress measurement approximately 20 min after the end of the TSST/Placebo-TSST, thus 35 min after stress onset, during which cortisol levels should peak^54^. At each time point, participants rated their subjective stress level on a visual analog scale, which was anchored with 0 and 100, and provided cortisol samples. Saliva samples were taken using Salivettes (Sarstedt, Germany) and kept at approximately − 18 °C until analysis. The cortisol samples were analyzed at the biochemical lab of the departments of Cognitive Psychology and Genetic Psychology, Ruhr University Bochum. Inter- and intraassay coefficients of variance were below 5%.

#### Structured clinical interview

To classify participants as individuals with risky or unproblematic gaming behavior, the structured clinical interview Assessment of Internet and Computer Game Addiction (AICA-SKI: IBS)^57^ was used, which assesses the DSM-5 criteria for gaming disorder^50^. Each of the nine criteria is rated regarding its occurrence in the last 12 months on a six-point Likert scale ranging from 0 (*not applicable*) to 5 (*very applicable*), with a rating of 4 or 5 indicating the criterion is met. The interviews were conducted by A.M.S., T.A.T., S.B., and N.K.E., who were trained and regularly supervised by A.M. and S.SL.

#### Ten-item Internet Gaming Disorder Test

Self-reported symptom severity of gaming disorder was measured with the Ten-Item Internet Gaming Disorder Test (IGDT-10)^58^, which is based on the DSM-5 criteria for gaming disorder. Each criterion is covered by one item, with exception of the last criterion, which is captured by two items. Participants’ ratings on the response scale (*never*,* sometimes*,* often*) are dichotomized, with the response option *often* indicating the criterion is fulfilled. With regard to criterion nine, it is considered met if at least one of the two items is answered with *often.* The resulting sum score ranges from 0 to 9.

#### Barratt Impulsiveness Scale

Impulsiveness was measured with the German short version of the Barratt Impulsiveness Scale (BIS-15)^59,60^. The scale consists of 15 items and comprises the three subscales non-planning, motor, and attentional impulsivity. Each item is rated on a 4-point Likert scale, ranging from 1 (*never/rarely*) to 4 (*almost always/always*). For the analyses, an overall sum score was used.

#### Go/No-Go Task

We used a Go/No-Go Task with gaming-related and control pictures to assess gaming-specific inhibitory control. The gaming-related pictures showed starting pages of different video games (distal cues, for more information see Diers et al.^61^) on the two devices that participants indicated as preferred devices for gaming, while the control pictures depicted different everyday items (e.g., pencil, keys…).

In this task, participants were required to react as fast as possible to go stimuli but withhold their response when a no-go stimulus was displayed. Which stimulus category (gaming vs. control) was defined as go or no-go stimulus changed between blocks and the order was counterbalanced across participants. The task consisted of 16 blocks with 20 trials and each block comprised ten gaming-related pictures and ten control pictures, which were displayed for 500 ms each in a randomized order. In case of incorrect responses, omissions, or too slow responses, a feedback screen was presented for 1000 ms. Otherwise, an intertrial interval (ITI) of 1000 ms consisting of a blank screen followed. To analyze gaming-specific inhibitory control, we calculated the number of commission errors for trials which required to inhibit responses towards the gaming stimulus^62,63^. Hence, the number of commission errors reflects the total number of responses towards the gaming-stimulus across trials in which the gaming-stimulus was defined as no-go stimulus, sometimes also referred to as ‚false alarms‘.

#### Additional measures of gaming-related symptoms, psychological distress, and cognitive abilities

Besides the tasks and instruments used to test our hypotheses, a number of additional measures was chosen to provide a more detailed description of our sample regarding further gaming-related variables, psychological distress, and cognitive abilities. Additionally to the IGDT-10, which captures the DSM-5 criteria for gaming disorder, we used the Assessment of Criteria for Specific Internet-use Disorders (ACSID-11)^64^, a screening instrument that is based on the ICD-11 criteria for disorders due to addictive behaviors^7^. (Potential) addictive behaviors captured by the ACSID-11 are gaming, online shopping, online pornography, social networks, and online gambling. The 11 items of the questionnaire cover the ICD-11 criteria impaired control, increased priority, and continuation/escalation despite negative consequences (with three items each), plus additional items assessing functional impairment and marked distress. Each item is answered on a two-part response scale, which captures both the frequency (0 = *never*, 1 = *rarely*, 2 = *sometimes*, 3 = *often*) and intensity (0 = *not at all intense*, 1 = *rather not intense*, 2 = *rather intense*, 3 = *intense*) of the symptoms. For our analyses, a mean score for gaming based on the frequency response scale of the questionnaire was calculated. Additionally, a mean frequency score for shopping was computed to check for potential group differences given that we used shopping-related pictures in our PIT paradigm.

In addition to gaming-related symptoms, habitual use of gaming was assessed with the Self-Report Habit Index (SRHI)^65^. The 12 items of the questionnaire were recoded, so that higher values indicated stronger habitual use of gaming, and summarized to a mean score. For measuring general psychological distress, the Brief Symptom Inventory (BSI-53)^66^ was used, whereby the item assessing suicidal tendency was removed, as acute suicidal ideation constituted an exclusion criteria and was hence assessed in the telephone screening. For the purpose of our study, we decided to calculate the global severity index, which is based on a mean score of all items. Finally, we measured logical thinking abilities, using part four of the German intelligence test battery Leistungsprüfsystem (Performance assessment system, LPS)^67^, which required participants to detect the logical mistake in 40 sequences of numbers and/or letters. As indicator for logical reasoning, the number of correct responses, ranging from 0 to 40, was used.

### Statistical analysis

To assess the effectiveness of the stress induction, mixed analyses of variance (ANOVA) with time as within-subjects factor and group (control, risky) and condition (TSST, Placebo-TSST) as between-subjects factors were computed separately for cortisol levels and subjective stress ratings. For both ANOVAs, we had to exclude participants due to missing values. This concerned one participant for the subjective stress response and three participants for the cortisol response.

The effects of group and condition as between-subjects factor and time (block 1–4) as within-subjects factor on response choice of the gaming-related reward during instrumental training were analyzed with a mixed ANOVA. Finally, mixed ANOVAs were used to analyze the effects of the between-subjects factors condition, group, and awareness (no, yes) and the within-subjects factors stimulus (gaming, shopping, neutral) and devaluation (pre, post) on response choice of the gaming-related reward during the transfer phase. The sample used for this analysis only included 133 participants, as one participant accidently skipped the devaluation procedure and one participant displayed missing values in the PIT-phase after devaluation. Mixed ANOVAs were chosen as they offer the possibility to test interactions between multiple within- and between-subject factors. The analyses were rerun excluding cortisol non-responders from the TSST group and cortisol responders from the placebo group. Following the criterion of Miller et al.^68^, individuals were considered as cortisol non-responders if they showed an increase in cortisol levels of less than 1.5 nmol/l. The same cutoff was used to identify cortisol responders in the placebo group. The difference in cortisol concentrations from T2 to T4 was chosen for identifying responders and non-responders. We decided to use T2 and not T1 to avoid confounding effects of potential cortisol increases during Pavlovian training.

To assess the effects of symptom severity, stress response, and the moderating effects of impulsivity and inhibitory control on the magnitude of the gaming PIT effect, hierarchical moderated regressions were computed. As indicator for the stress response, the cortisol difference between T2 and T4 was used. All predictors were centered at their mean before being entered in the regression analyses^69^.

The assumptions of all statistical procedures applied were checked. In case of violation of the sphericity assumption, Greenhouse-Geisser (if Greenhouse-Geisser ε < 0.75) or Huynh-Feldt (if Greenhouse-Geisser ε > 0.75) adjusted degrees of freedom were reported. The significance level was set at 0.05. If significant main or interaction effects were found, post hoc analyses with Bonferroni corrected *t*-tests were conducted. Depending on the statistical procedure used, effect sizes are reported in partial eta squared ($\upeta_{\rm{p}}^{2}$) or R-squared (R^2^).

Following the suggestions by Field and Wilcox^70^, we provide additional robust estimates for the multiple regression analyses and the ANOVAs. As robust estimates for the multiple regression, we report bootstrapped standard errors and confidence intervals for the unstandardized regression coefficients, which were obtained using bias-corrected and accelerated bootstrapping with 1,500 iterations. Regarding the mixed ANOVAs, the most relevant main effects and two-way interactions were further verified using robust ANOVAs based on 20% trimmed means^70,71^. These analyses were only conducted for the whole sample and not for the subsample in which the conditions were corrected for cortisol responses. For both the regression analyses and the ANOVAs, results were considered as reliable, if the results of the robust methods were in line with those of the conventional methods^70^. The mixed ANOVAs on trimmed means were computed with R (version 4.3.3), using the bwtrim function of the package WRS2^72^, all other statistical analyses were computed using IBM SPSS Statistics (version 29).

### Power analysis

An a priori power analyses, which was conducted using G*Power (3.1.9.2)^73,74^, indicated that a sample size of *N* = 128 was necessary to find a medium-sized effect of the stress induction compared to the control condition with a power of 0.80 when investigating four different groups of participants (risky/unproblematic use x TSST/Placebo-TSST). The selection of a medium-sized effect was based on our previous research ^e.g., 20^ and a pilot study^16^, in which we examined the effects of acute stress on cue-driven responding in a PIT paradigm.

### Transparency and openness

The study protocol was preregistered at OSF: https://osf.io/f27qw/?view_only=4bcea30152d54aab8d6c191e269cbe7d.

Besides the study presented here, the central project of the research unit, including the core battery, was also preregistered at OSF: https://osf.io/6x93n/?view_only=00fe0424a3ca4eda96eebd9bfc4efb4b.

We deviated from our preregistration in the questionnaires used to describe symptom severity, as a recent review did not support the use of the s-IAT for assessing gaming disorder due to its missing alignment with diagnostic manuals and limited evidence of its psychometric properties^75^. Instead, we relied on the IGDT-10 and ACSID-11 to measure symptom severity, which – though not included in the preregistration of this specific sub-project of the research unit – were preregistered as part of the overall project of the FOR2974.

## Results

### Sample characteristics

Participants were predominantly male, in their mid-twenties and had an average school education of 13 years (see Table 1). The group with risky gaming behavior and the control group did not differ regarding age and gender, which confirmed that the matching was successful. As per inclusion criteria, the participants with risky gaming behavior met more criteria in the clinical interview compared to the control participants. The validity of our group allocation was supported by individuals with risky gaming behavior indicating significantly higher levels of symptom severity in the self-report measures of gaming disorder. In addition, the individuals with risky gaming behavior reported significantly higher habit strength with regard to gaming as well as higher levels of impulsiveness and psychological distress.

**Table 1:** Descriptive statistics and group comparison.

Sample characteristics	Risky group (*n* = 68)	Control group (*n* = 67)	Group comparison	Cronbach’s α
Gender, *n* (%)			χ²(1) = 0.08, *p* = .78, φ = 0.02	
Female	9 (13.2)	10 (14.9)		
Male	59 (86.8)	57 (85.1)		
Age (years)	24.24 (4.61)	24.19 (3.69)	*t*(133) = 0.06, *p* = .95, *d* = 0.01	
BMI (kg/m^2^)	23.66 (3.51)	24.45 (3.38)	*t*(132) = − 1.31, *p* = .19, *d* = − 0.23	
Education (school years)	12.72 (0.81)	12.93 (0.26)	*t*(81.43) = − 1.99, *p* = .05, *d* = − 0.34	
Number of criteria in clinical interview (AICA-SKI IBS)	3.00 (1.00)	0.33 (0.47)	***t*(94.14) = 19.77**, ***p*** **< .001**, ***d*** **= 3.42**	
Symptom severity gaming (IGDT-10)	1.48 (1.52)	0.59 (0.89)	***t*(107.72) = 4.10**, ***p*** **< .001**, ***d*** **= 0.71**	0.77
Symptom severity gaming (ACSID-11)	0.96 (0.57)	0.51 (0.36)	***t*(112.26) = 5.46**, ***p*** **< .001**, ***d*** **= 0.95**	0.88
Symptom severity shopping (ACSID-11)	0.13 (0.29)	0.17 (0.31)	*t*(124) = − 0.74, *p* = .46, *d* = − 0.13	0.90
Habit strength gaming (SRHI)	3.25 (0.68)	2.46 (0.76)	***t*(123.81) = 6.20**, ***p*** **< .001**, ***d*** **= 1.09**	0.89
Impulsiveness (BIS-15)	33.55 (6.23)	31.27 (5.40)	***t*(132) = 2.27**, ***p*** **= .03**, ***d*** **= 0.39**	0.77
Inhibitory control (gaming-related CE in Go/No-Go Task)	3.87 (3.80)	3.53 (2.61)	*t*(129) = 0.58, *p* = .56, *d* = 0.10	
Psychological distress (BSI-53, GSI)	0.52 (0.34)	0.35 (0.24)	***t*(118.87) = 3.35**, ***p*** **= .001**, ***d*** **= 0.58**	0.92
Logical thinking abilities (LPS)	30.99 (3.34)	31.80 (3.19)	*t*(131) = − 1.44, *p* = .15, *d* = − 0.25	

All values are displayed as means and standard deviations (SD) in brackets if not otherwise specified. Due to missing values, sample sizes for the descriptive statistics ranged from 65 to 68 in the risky group, and from 61 to 67 in the control group. To test for group differences, χ^2^-test were conducted for gender, while two-sided *t*-tests were performed for continuous variables. For *t*-tests, Welch’s correction was applied if the Levene-test indicated non-homogeneous variances.

Significant differences are highlighted in bold.

*AICA-SKI IBS* Assessment of Internet and Computer Game Addiction-Structured Clinical Interview, *ACSID-11* Assessment of Criteria for Specific Internet-use Disorders, *IGDT-10* Ten-Item Internet Gaming Disorder Test, *SRHI* Self-Report Habit Index, *BIS-15* Barratt Impulsiveness Scale – short version, *CE* commission errors, *BSI-53* Brief Symptom Inventory, *GSI* Global Severity Index, *LPS* Leistungsprüfsystem [Performance assessment system].

Awareness of the experimental contingencies, i.e., the knowledge, which stimulus predicted the gaming-related pictures and which stimulus predicted the shopping-related pictures, was acquired by 72.6% of the participants. There were no differences in awareness between groups (χ^2^(1) = 0.02, *p* = .89, φ = 0.01) or conditions (χ^2^(1) = 0.83, *p* = .36, φ = 0.08).

### Subjective stress and cortisol responses to the stress induction

Concerning subjective stress, the main effects of time (*F*(2.10, 272.37) = 91.48, *p* < .001, $\upeta_{\rm{p}}^{2}$ = 0.41) and condition (*F*(1, 130) = 10.67, *p* = .001, $\upeta_{\rm{p}}^{2}$ = 0.08) were qualified by an interaction between condition and time (*F*(2.10, 272.37) = 36.27, *p* < .001, $\upeta_{\rm{p}}^{2}$ = 0.22). While the conditions did not differ significantly at T1 (*p* = .88) and T2 (*p* = .91), individuals in the TSST condition reported significantly more subjective stress than those in the control condition at T3 (*p* < .001) and T4 (*p* = .007).

**Fig. 3:**
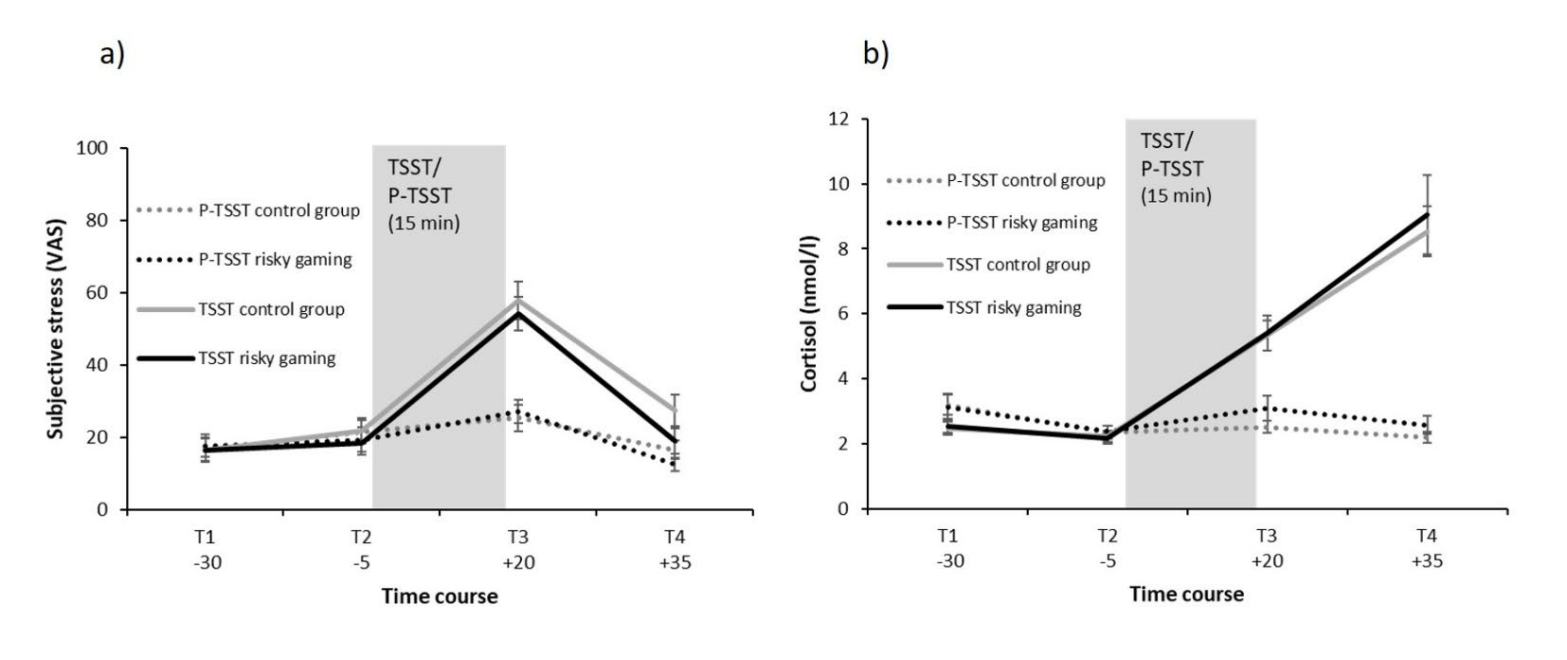
Stress response by condition and group. The figures display (**a**) the subjective stress and (b) cortisol response in the stress (TSST) and placebo (P-TSST) condition in individuals with risky gaming behavior and control participants. Subjective stress was rated on a visual analogue scale (VAS) from 0 to 100. The times are specified relative to stress onset. (**a**) P-TSST control group: *n* = 34; P-TSST risky gaming: *n* = 33; TSST control group: *n* = 33; TSST risky gaming: *n* = 34. (**b**) P-TSST control group: *n* = 34; P-TSST risky gaming: *n* = 33; TSST control group: *n* = 32; TSST risky gaming: *n* = 33. Means and standard errors of the means are presented. *TSST* Trier Social Stress Test, *P-TSST* Placebo Trier Social Stress Test.

Similarly for cortisol, the main effects of time (*F*(1.44, 184.17) = 62.44, *p* < .001, $\upeta_{\rm{p}}^{2}$ = 0.33) and condition (*F*(1, 128) = 39.73, *p* < .001, $\upeta_{\rm{p}}^{2}$ = 0.24) were qualified by a significant condition by time interaction (*F*(1.44, 184.17) = 75.71, *p* < .001, $\upeta_{\rm{p}}^{2}$ = 0.37). Individuals in the TSST condition did not differ in their cortisol levels from participants in the control condition at T2 (*p* = .36), but showed significantly higher cortisol levels at T3 (*p* < .001) and T4 (*p* < .001). Additionally, an unexpected difference between the conditions was observed at T1 (*p* = .03), with individuals in the control condition displaying significantly higher cortisol levels compared to participants in the stress condition. In contrast to subjective stress, which peaked directly after the stress induction (see Fig. 3a), cortisol levels increased slowly after the stress induction and were highest at T4, i.e., around 20 min after the TSST (see Fig. 3b). A significant main effect of group or significant interactions with group did emerge neither for cortisol nor the subjective stress ratings (all *F*s ≤ 1.56, all *p*s ≥ 0.21), thus, participants with risky gaming behavior did not differ from control participants in their stress responses.

Results of the robust ANOVAs supported the significant condition*time interaction for cortisol and subjective stress (see Table S2 in the supplemental material).

According to the 1.5 nmol/l criterion, 12.3% individuals were classified as non-responders in the TSST group and 10.4% as cortisol responders in the placebo group. The adjusted conditions did not significantly differ regarding the percentage of individuals with risky gaming behavior (TSST adjusted: 47.4%, placebo adjusted: 46.7%; χ^2^(1) = 0.01, *p* = .94, φ = 0.01).

As the use of oral contraceptives as well as the phase of the menstrual cycle can influence the cortisol response towards acute stress^76^, we tested whether these variables differed between groups and conditions. Significant differences were found neither for group nor for condition (for more information see Table S1 in the supplementary material).

### Effect of risky gaming and stress on general responding for gaming-related rewards

A mixed ANOVA on response choice during instrumental training yielded a significant main effect of time (*F*(2.96, 388.04) = 10.82, *p* < .001, $\upeta_{\rm{p}}^{2}$ = 0.08) and group (*F*(1, 131) = 7.66, *p* = .006, $\upeta_{\rm{p}}^{2}$ = 0.06), however no significant main or interaction effect of condition (all *F*s ≤ 0.83, all *p*s ≥ 0.47). The main effect of time indicated that the choice of the gaming-related reward decreased from the first block to the subsequent blocks of the instrumental training (all *p*s < 0.001), while the main effect of group reflected higher choice of the gaming-related reward in individuals with risky gaming behavior compared to the control group.

Results of the robust mixed ANOVAs conducted separately for the factors group and time and the factors condition and time supported the significant main effects of group and time (see Table S3 in the supplemental material).

When rerunning the analyses after excluding cortisol non-responders in the TSST condition and responders in the Placebo condition, the main effect of time (*F*(3, 339) = 10.03, *p* < .001, $\upeta_{\rm{p}}^{2}$ = 0.08) and group (*F*(1, 113) = 3.99, *p* = .048, $\upeta_{\rm{p}}^{2}$ = 0.03) remained significant. Again, no significant effect of condition emerged (all *F*s ≤ 0.90, all *p*s ≥ 0.44).

### Effect of risky gaming, stress, and devaluation on stimulus-associated responding for gaming-related rewards

The mixed ANOVA with response choice during the transfer phase as dependent variable revealed a main effect of stimulus (*F*(1.38, 172.83) = 62.66, *p* < .001, $\upeta_{\rm{p}}^{2}$ = 0.33), which was qualified by a stimulus by awareness interaction (*F*(1.38, 172.83) = 26.97, *p* < .001, $\upeta_{\rm{p}}^{2}$ = 0.18). Individuals who were aware of the experimental contingencies showed a gaming PIT effect, i.e., pressed the gaming-related key significantly more often in CS^Gaming^ trials compared to trials in which the neutral stimulus was presented (*p* < .001; for descriptive results see supplements, Table S4). Additionally, there was a significant difference between the CS^Shopping^ and the neutral stimulus, with the first one being associated with a significant lower choice of the gaming-related key (*p* < .001). In contrast, the response behavior of unaware participants was not influenced by the stimulus presented (all *p*s ≥ 0.27). Participants with risky gaming behavior showed a greater preference for the gaming-related reward compared to the control group, as evidenced by a significant main effect of group (*F*(1, 125) = 5.96, *p* = .02, $\upeta_{\rm{p}}^{2}$ = 0.05).

**Fig. 4:**
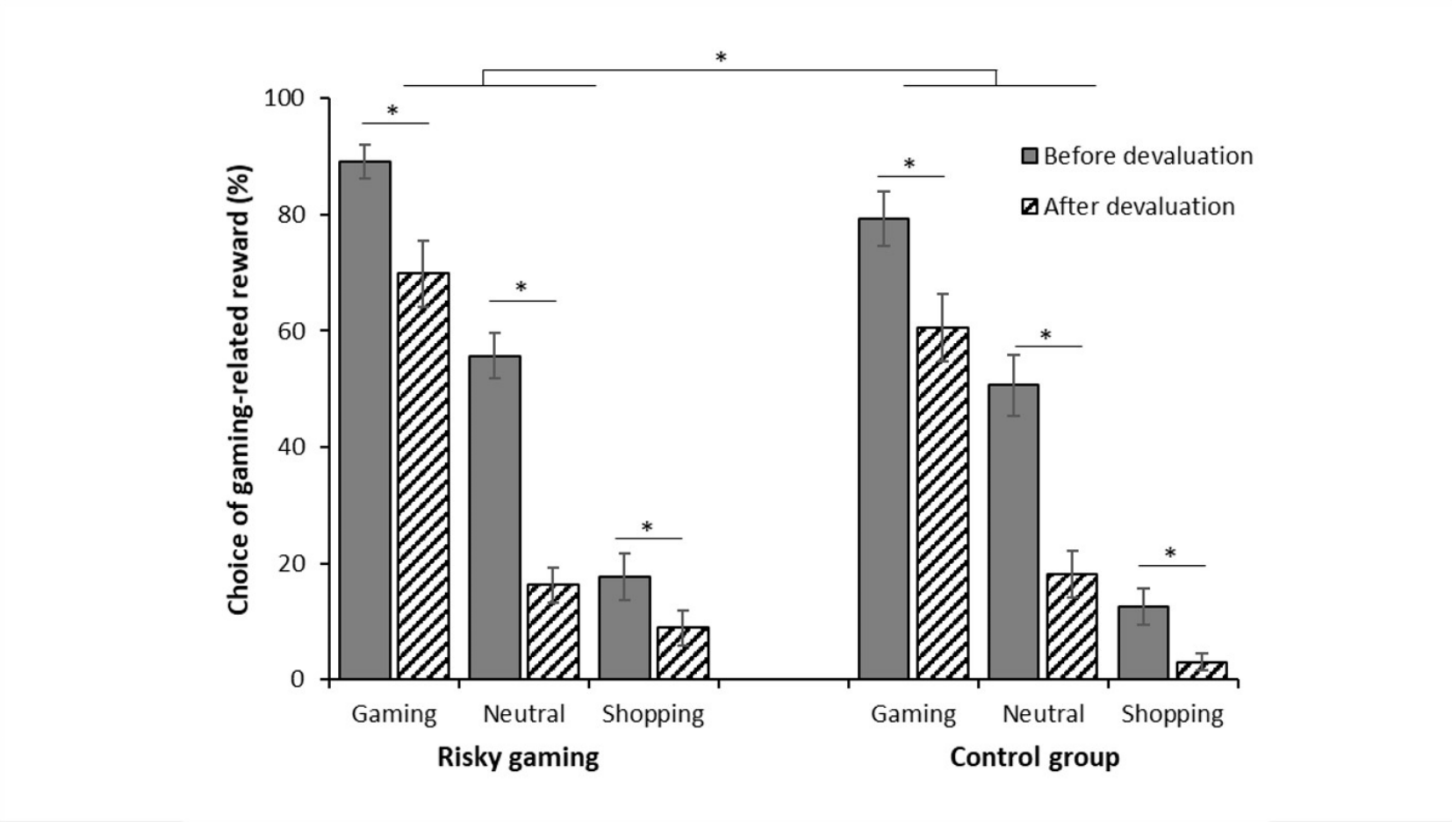
Choice of the gaming-related reward before and after devaluation by group – Aware participants. Percentage choice of the gaming-related response (compared to the shopping-related response) after presentation of the gaming-related stimulus, the neutral stimulus (gray square), and the shopping-related stimulus before and after devaluation in aware participants with risky gaming behavior (*n* = 48) and aware control participants (*n* = 49). *p < .05.

A main effect of devaluation (*F*(1, 125) = 68.62, *p* < .001, $\upeta_{\rm{p}}^{2}$ = 0.35) indicated a general decrease in response choice for the gaming-related reward after devaluation (see Fig. 4). This decrease was largest in presence of the neutral stimulus, as indicated by a significant devaluation by stimulus interaction (*F*(2, 250) = 24.29, *p* < .001, $\upeta_{\rm{p}}^{2}$ = 0.16). The expected devaluation by group interaction was not significant (*F*(2, 125) = 0.01, *p* = .92, $\upeta_{\rm{p}}^{2}$ < 0.001).

**Fig. 5:**
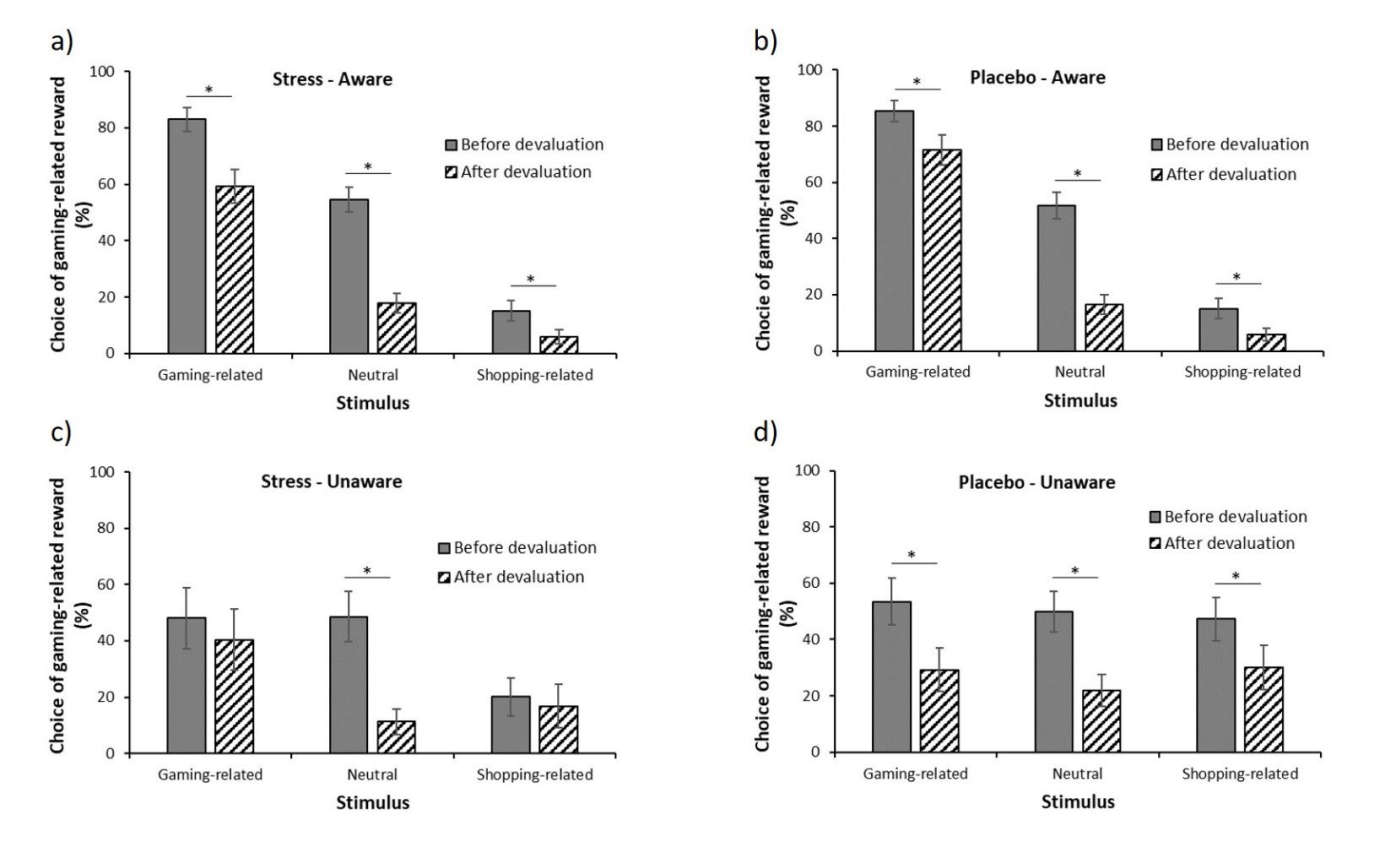
Choice of the gaming-related reward before and after devaluation by condition and awareness. Percentage choice of the gaming-related response (compared to the shopping-related response) after presentation of the gaming-related stimulus, the neutral stimulus (gray square), and the shopping-related stimulus before and after devaluation for (**a**) aware individuals in the TSST condition (*n* = 50), (**b**) aware individuals in the placebo condition (*n* = 47), (**c**) unaware individuals in the TSST condition (*n* = 15), (**d**) unaware individuals in the placebo condition (*n* = 21). Means and standard errors of the means are presented. * *p* < .05.

In addition, a significant devaluation by stimulus by condition by awareness interaction emerged (*F*(2, 250) = 3.36, *p* = .04, $\upeta_{\rm{p}}^{2}$ = 0.03). Post-hoc tests indicated, contrary to our expectations, that stress did not reduce the effect of the devaluation in aware participants. Thus, aware participants in the TSST as well as placebo condition reduced their overall responding for the gaming-reward after the devaluation (all *p*s < 0.01, see Fig. 5a, b), but still showed a PIT effect after devaluation. In contrast, while unaware participants in the placebo condition also decreased their overall responding (all *p*s < 0.002, Fig. 5d), unaware participants in the TSST condition did not show this reduction in response to the CS^Gaming^ (*p* = .48) and the CS^Shopping^ (*p* = .48, see Fig. 5c).

All other main or interaction effects of condition were not significant (all *F*s ≤ 3.05, all *p*s ≥ 0.08). To validate the findings, robust ANOVAs were calculated separately for responding before and after the devaluation. The main effects of condition, group, and awareness and their interaction with stimulus were tested in three separate analyses. While the main effect of stimulus and the stimulus*awareness effect could be replicated both before and after the devaluation, the main effect of group did not reach significance (see Table S5 in the supplemental material).

Again, the analyses were rerun after adjusting the TSST and placebo condition for cortisol non-responders and cortisol responders, respectively. All significant effects could be replicated. In addition, a stimulus by awareness by condition interaction emerged (*F*(1.38, 148.67) = 4.18, *p* = .03, $\upeta_{\rm{p}}^{2}$ = 0.04), however, not in the way we expected. While stimulus-dependent responding did not differ between the stress and control condition in aware participants (all *p*s > 0.21), unaware participants displayed decreased responding towards the CS^Shopping^ in the stress compared to the control condition (*p* = .001).

### Predictors for the magnitude of the PIT effect after devaluation

Bivariate correlations indicated a significant positive association between symptom severity of gaming disorder and the magnitude of the gaming PIT effect after devaluation, however not with the magnitude of the PIT effect before devaluation (see Table 2). Neither inhibitory control, impulsiveness nor the cortisol response were significantly correlated with the magnitude of the gaming PIT effect after devaluation.

**Table 2:** Bivariate correlations of PIT-related variables with addiction-related variables, cortisol response and gender in the whole sample. *n* = 123. Bivariate Pearson correlations were computed. *IGDT-10* Ten-Item Internet Gaming Disorder Test; Cortisol response was computed as difference (post–pre) in cortisol concentrations between T2 and T4, *T2* second time point of stress measurement, *T4* fourth time point of stress measurement, *CE* commission errors, *BIS-15* Barratt Impulsiveness Scale – short version. *p < .05.

Variable	(1)	(2)	(3)	(4)	(5)	(6)	(7)
(1) Magnitude of gaming PIT effect after devaluation							
(2) Magnitude of gaming PIT effect before devaluation	0.45*						
(3) Awareness	0.34*	0.38*					
(4) Symptom severity (IGDT-10)	0.20*	0.03	0.19*				
(5) Cortisol response (T4-T2)	− 0.13	− 0.04	0.09	0.003			
(6) Inhibitory control (gaming-related CE in Go/No-Go Task)	− 0.07	− 0.01	− 0.23*	− 0.13	− 0.06		
(7) Impulsiveness (BIS-15)	0.003	− 0.05	− 0.06	0.17	0.01	− 0.01	
(8) Gender	0.20*	0.21*	0.04	0.20*	− 0.21*	− 0.03	0.01

Hierarchical linear regressions were computed to analyze the effects of symptom severity, cortisol response and the moderating effects of impulsiveness and inhibitory control on the magnitude of the gaming PIT. Due to their significant correlations with the magnitude of the gaming PIT effect after devaluation, gender, the magnitude of the gaming PIT effect before devaluation, and awareness were entered as control variables in the first step. The PIT effect before devaluation (*B* = 0.42; ß = 0.35, *p* < .001) and awareness (*B* = 19.25, ß = 0.20, *p* = .02) revealed to be significant predictors. The effects were robust when applying bootstrapping: The bootstrapped 95%-confidence intervals for the unstandardized regression coefficient did not include zero for both the PIT effect before devaluation (95%-CI for *B* = [0.19; 0.66]) and awareness (95%-CI for *B* = [2.74; 33.73]). None of the predictors added in the next steps, i.e., symptom severity, cortisol response, inhibitory control, impulsiveness, and the interaction terms between inhibitory control/impulsiveness and the cortisol response, were significant. The results of the last step are presented in Table 3. While the PIT effect before devaluation remained significant in the last step, the bootstrapped confidence interval for awareness included zero, proving its significant *p* value in the last step to be unreliable.

Given the relevance of awareness for the PIT effect, the analyses were rerun, restricting the sample to aware participants. This time, the cortisol response, added in the third step, emerged as additional significant predictor (*B* = -1.62; ß = − 0.24, *p* = .02), which was also supported by the bootstrapping results (95%-CI for *B* = [-2.84; − 0.49]). However, in contrast to our expectation, a higher cortisol response was associated with a reduced PIT effect after devaluation. None of the predictors added in the next steps, i.e., inhibitory control, impulsiveness, and the interaction terms between inhibitory control/impulsiveness and the cortisol response, were significant. The results of the last step are presented in Table 4.

**Table 3:** Hierarchical regression results for the magnitude of the gaming PIT effect after devaluation – whole sample.

Variable	*B*	95% CI for *B*^a^	*SE* *B* ^a^	β	*t*	*p*
Step 1						
Gender	7.94	[− 10.74; 26.50]	10.06	0.07	0.77	0.45
Magnitude of gaming PIT effect before devaluation	**0.44**	**[0.20; 0.70]**	**0.12**	**0.37**	**4.14**	**< 0.001**
Awareness	17.15	[− 1.01; 34.71]	9.25	0.18	2.02	0.046
Step 2						
Symptom severity	4.49	[− 0.68; 10.68]	2.89	0.14	1.66	0.10
Step 3						
Cortisol response	− 0.77	[− 2.05; 0.69)	0.68	− 0.10	− 1.11	0.27
Step 4						
Inhibitory control	− 0.04	[− 1.88; 1.92]	1.06	− 0.003	− 0.04	0.97
Impulsiveness	0.08	[− 1.10; 1.02]	0.59	0.01	0.13	0.90
Step 5						
Cortisol response x inhibitory control	0.17	[− 0.25; 0.63]	0.24	0.05	0.62	0.54
Cortisol response x impulsiveness	− 0.09	[− 0.33; 0.15]	0.11	− 0.06	− 0.77	0.45
*R*^*2*^	0.29					

*n* = 123. Regression coefficients of the final model, after step 5, are presented. *B* refers to the unstandardized regression coefficients, β to the standardized regression coefficients.

Significant effects are highlighted in bold.

Cortisol response was computed as difference (post–pre) in cortisol concentrations between T2 and T4.

^a^Confidence intervals and standard errors for the unstandardized regression coefficients were derived by bias-corrected and accelerated bootstrapping with 1500 iterations.

**Table 4:** Hierarchical regression results for the magnitude of the gaming PIT effect after devaluation – aware participants.

Variable	*B*	95% CI for **B**^a^	*SE B* ^a^	β	t	*p*
Step 1						
Gender	14.99	[− 7.03; 36.10]	11.16	0.13	1.27	0.21
Magnitude of gaming PIT effect before devaluation	**0.38**	**[0.09; 0.64]**	**0.14**	**0.27**	**2.73**	**0.01**
Step 2						
Symptom severity	4.40	[− 0.66; 11.02]	3.13	0.16	1.53	0.13
Step 3						
Cortisol response	− **1.77**	**[**− **3.49;** − **0.29]**	**0.74**	**− 0.26**	− **2.19**	**0.03**
Step 4						
Inhibitory control	0.70	[− 2.29; 3.61]	1.56	0.05	0.45	0.65
Impulsiveness	0.47	[− 0.87; 1.55]	0.68	0.07	0.72	0.47
Step 5						
Cortisol response x inhibitory control	− 0.16	[− 0.70; 0.38]	0.30	− 0.05	− 0.45	0.66
Cortisol response x impulsiveness	− 0.12	[− 0.36; 0.18]	0.12	− 0.09	− 0.93	0.36
*R*^*2*^	0.24					

*n* = 89. Regression coefficients of the final model, after step 5, are presented. *B* refers to the unstandardized regression coefficients, β to the standardized regression coefficients.

Significant effects are highlighted in bold.

Cortisol response was computed as difference (post–pre) in cortisol concentrations between T2 and T4.

^a^Confidence intervals and standard errors for the unstandardized regression coefficients were derived by bias-corrected and accelerated bootstrapping with 1500 iterations.

## Discussion

This study aimed to investigate instrumental responding in the presence of gaming-related cues and the potential shift to habitual behavior under stress in individuals with risky gaming behavior. For this purpose, we applied a PIT paradigm with gaming-related cues in combination with a stress induction and devaluation procedure. In line with previous studies^20,22^, a gaming-related PIT effect was found in individuals who had successfully learned the experimental contingencies during Pavlovian training. While Qin et al.^22^, who used a similar version of the PIT paradigm, observed increased PIT effects in individuals with gaming disorder, we did not find significant group differences with regard to the PIT effect. Yet this may be attributed to the fact that we studied individuals with risky but not pathological gaming behavior. However, we found a group difference with regard to general response choice. Compared to the control group, individuals with risky gaming behavior showed a greater preference for the gaming-related reward during instrumental training and the transfer phase, though only the first effect was supported by the robust analyses. Hence, gaming-related rewards seem to be of increased value not only for individuals who have developed a gaming disorder^22^, but already for individuals with risky gaming behavior. However, the effect size was only small to medium, thus, future studies are needed to support this finding.

While our devaluation successfully decreased responding towards all stimuli, the gaming-related stimulus continued to increase responding for the gaming-related reward, which points to a habitual character of the gaming PIT effect. Unexpectedly, such habitual responding towards gaming-related cues was observed not only in individuals with risky gaming behavior but also in the control group. A comparable effect has been observed for food-related rewards, with healthy individuals continuing to respond towards food-associated cues despite satiation^77^. In this regard, it should be emphasized that habits are not problematic per se^78,79^ as they allow to facilitate a regularly occurring behavior^79^ and may, thus, also be observed in the absence of problematic behavior. However, we found a significant correlation between symptom severity and the magnitude of the PIT effect after devaluation, which could not be explained with greater PIT effects per se, considering the non-significant association between symptom severity and the magnitude of the PIT effect *before* devaluation. This observation together with the higher level of self-reported gaming habits in individuals with risky gaming behavior points towards a stronger tendency towards habitual behavior in this group and lends some support for the role of habits in the development and maintenance of gaming disorder. If individuals tend to automatically engage in gaming when encountering gaming-related triggers, not only can the behavior easily escalate but it would also be difficult to change. These habits might become especially detrimental when meeting individual vulnerability factors associated with addictions and/or interacting with other addiction-related processes, finally resulting in compulsive behavior. For example, individuals with risky gaming behavior may attribute greater incentive salience^80^ to gaming-related stimuli, which may influence their tendency to learn associations between neutral and gaming-related cues. In fact, in line with a previous study^20^, we found a positive association between awareness and symptom severity. Furthermore, in our sample, individuals with risky gaming behavior showed significantly higher levels of trait impulsiveness, as measured with the Barratt Impulsiveness Scale. Although trait impulsivity was not directly linked to the size of the PIT effect, it may be one factor influencing whether habit-like PIT effects lead to a problematic usage. For example, high trait impulsivity may impede individuals to plan ahead more carefully to avoid potential triggers (e.g., remove the gaming equipment when studying) or to stop playing when other tasks need to be done.

Contrary to our assumptions, we did not find that stress increased responding for gaming-related rewards or decreased the effect of the devaluation procedure in individuals who were aware of the experimental contingencies. Instead, stress and, more specifically, the cortisol response was negatively associated with the size of the PIT effect after devaluation in aware participants. This unexpected negative effect of stress on cue-related responding might be explained with increased response inhibition, a cognitive function that had been shown – at least in healthy populations – to be enhanced under acute stress^81^. Consequently, individuals might have been better able to control automatic responses in the presence of gaming-related cues after devaluation. Moreover, there is some, albeit inconsistent, evidence, that gaming itself – also depending on the type or content of the game – can induce stress-like responses^82,83^. Hence, individuals may have gotten used to function under stress, which may be one explanation for the negative association between stress and the size of the PIT effect in aware participants. However, in light of the small effect size of the stress effect, the practical relevance of this finding may be limited.

Our findings are difficult to align with both the view that individuals deliberately turn to gaming to cope with stress^84^, which should have increased the value of the gaming-related reward^4^, resulting in a greater choice of the gaming-related response *before* devaluation, and the assumption that individuals tend to habitual behavior under stress, which should have led to a reduced devaluation effect under stress. Although our findings are in contrast to earlier findings of a stress-induced shift to habitual behavior^25^, they are in line with two recent studies that failed to find a stress-induced shift to habitual decision making in individuals with gambling disorder^85,86^. In light of our findings, the view that stress is a general trigger for problematic gaming may be too narrow. Instead, the influence of stress on problematic gaming may depend on further variables, for example the usage motives individuals have. According to a cluster analysis by Billieux et al.^87^, not all individuals with problematic gaming behavior play to escape negative emotions but one group was rather characterized by high achievement motives. The effect of stress may differ among those groups and future studies may hence benefit from exploring usage motives as a moderating variable.

Unexpectedly, individuals in the stress condition who were unaware of the experimental contingencies did not significantly reduce performance of the gaming response in presence of the gaming and shopping stimulus after devaluation. The pattern of response choice before devaluation indicates that these individuals, despite lacking explicit awareness, might have acquired some implicit knowledge of the stimulus-outcome association. Therefore, stress may have promoted habitual behavior in our study however only towards more implicitly learnt cues. If individuals are not aware of the stimulus-outcome associations they have formed, exerting inhibitory control in the presence of such triggers may be more difficult, hence increasing the likelihood to behave habitually under stress. Based on our findings, there may be two pathways towards habitual responding. One pathway may concern habitual responding towards explicitly learnt cues and may be linked to symptom severity, which seems to influence not only habitual responding but also the tendency to learn associations between neutral cues and addiction-related content. The other pathway towards habitual responding may be triggered by stress and may not require explicit awareness of the stimulus-outcome associations, but instead present a reaction towards implicitly learnt cues. Hence, when considering habitual responding, it may be relevant to distinguish between explicitly and implicitly learnt cues.

However, in light of the small number of unaware participants in the stress condition (*n* = 15) and the multiple tests conducted, future studies are needed to ensure the reliability of the finding. Furthermore, decreased devaluation effects in individuals who lack explicit contingency knowledge have already been described in a review by Hogarth^4^. Rather than interpreting it as a sign for habitual behavior, Hogarth assumed task disengagement due to general cognitive and motivational deficits to be responsible for this effect.

Considering potential stress effects on memory, it is important to note that stress did not impair retention or retrieval of the stimulus-outcome associations in our study as evidenced by comparable awareness rates between conditions. This finding is consistent with the results of a meta-analysis by Shields et al.^88^, who reported no significant effects of stress on memory if stress affected both the postencoding and the retrieval phase.

In sum, our results implied a general tendency toward habitual behavior in the presence of gaming-related cues, which supports the relevance of habits in the development and maintenance of gaming disorder. In addition, preliminary findings suggest that stress may promote a shift towards stronger habitual behavior in participants without explicit knowledge of the reward-related experimental contingencies.

### Strengths and limitations

As far as we know, this experiment is the first to study habitual behavior and the potential moderating influence of stress in the context of problematic gaming. Furthermore, by focusing on individuals with risky but not pathological gaming behavior we did not only study a group which has received little attention in previous research, but were also able to demonstrate that habitual behavior can already be observed in individuals with risky gaming behavior. With regard to the methodological approach, our study has two major strengths. First, not only were our groups matched with regard to age and gender, but we also ensured that participants in both groups had gaming experience. This allowed for a very rigorous test of the effects of risky use. Second, the diagnosis of risky gaming behavior was ensured by a clinical interview, and the validity of our group assignment was further supported by significant group differences on the self-report measures of gaming disorder.

However, when interpreting the findings of our study, there are also some limitations, which must be considered. First, our sample was not representative for all individuals with risky gaming behavior, as our participants were predominantly male and highly educated. The exclusion criteria applied due to the stress induction (e.g., BMI outside the range of 17–31 kg/m^2^ or use of medication known to influence the HPA axis) may have further narrowed the representativeness of our sample.

Second, although we used eight instead of four blocks of Pavlovian training as in a previous study^20^, around one third of our participants did not learn the stimulus-outcome associations. As this number is in line with our previous study, future studies should consider adapting the PIT paradigm to increase the percentage of aware participants, for example by including an explicit instruction at the beginning of the Pavlovian training, stating that the abstract stimuli shown have informative value for the subsequent pictures ^e.g., 77^. However, this approach may initiate a more intentional form of learning, which could differ from the more incidental way associations between neutral and addiction-related stimuli are learned in the natural environment^89^. Alternatively, a paradigm as described, for example, by Hogarth and Chase^27^ or Martinovic et al.^18^ may be used, in which the Pavlovian training phase is omitted and instead of experimentally conditioned stimuli, naturally conditioned cues, i.e., pictures related to the addictive behavior, are presented during the transfer phase.

A third limitation concerns our devaluation procedure. By informing participants that the maximum amount of the gaming voucher had been achieved, one may argue that we extinguished the response-outcome association rather than altered the value of the outcome ^90^. Yet, while reducing the reward value can be successfully achieved for food-related rewards by changing the taste or by consumption to satiety^24,25^, it is more difficult to realize with regard to gaming-related outcomes. An alternative devaluation procedure that has already been applied with regard to food-related^91^ and tobacco-related outcomes^27^ is the use of health warnings. A similar approach may be explored in the context of gaming, for example, by confronting participants with the negative consequences of excessive gaming behavior.

Fourth, we only assessed awareness of the stimulus-outcome associations operating during Pavlovian training, however did not test knowledge of the response-outcome association or outcome expectancies following devaluation. Hence, we cannot be sure if individuals performed the behavior despite being aware of its altered reward value. This is an important aspect for basic research given that the transfer from goal-directed to habitual and finally compulsive behavior is assumed a key process in the development of addictive behavior^6^. Compulsive behavior is described as the persistence of the behavior despite loss of value of the reinforcer^4^. A study by Gillan et al.^92^ provided evidence that insensitivity towards devaluation can emerge despite intact contingency awareness and correct expectancies regarding the devalued outcome^92^, hence paralleling compulsive behavior. However, the learning paradigm applied by Gillan et al.^92^ differed from the PIT task, as it did not involve separate instrumental and Pavlovian conditioning. Future studies are thus warranted to further understand the exact mechanisms underlying the PIT effect.

Fifth, many of the tasks applied in our study required individuals to focus their attention. Low performance is these tasks, like not acquiring awareness in the PIT paradigm or making many commission errors in the Go/No-Go task, may hence stem from individuals failing to pay the necessary attention, thereby bearing the risk of causing misleading correlations between the variables. For example, the negative association between awareness and the Go/No-Go task may be – at least partly – caused by impaired attention.

Finally, we provided additional robust estimates in our analyses, however, were not able to verify all results, as the WRS2 package used allows to conduct only a two-way mixed ANOVA, hence preventing us from testing interactions between more than two variables. Yet, we considered the advantages of the WRS2 package, especially its comprehensive documentation, to outweigh this limitation. Moreover, the two-way mixed ANOVA was sufficient to confirm one of the main results of our study, i.e., the presence of the PIT effect even after devaluation.

### Directions for future research

Risky gaming behavior, as investigated in our study, may reflect an early stage of the addiction process as described by the Interaction of Person-Affect-Cognition-Execution (I-PACE) model^5^. However, in a study by Scharkow et al.^93^, problematic gaming behavior appeared to be rather transient, with less than a third of individuals with initially problematic gaming behavior being classified as such two years later. The low stability of problematic gaming parallels findings from problematic gambling^94–96^. Thus, the observation of habitual behavior in individuals with risky gaming behavior does not necessarily imply that this is a mechanism relevant for the development of gaming disorder. Hence, longitudinal studies are needed to (a) explore the transition from risky to pathological gaming behavior and (b) investigate the association between habitual PIT effects and the development of gaming disorder.

Relatedly, compulsive drug seeking has been explained with a decreased ability to switch between the habitual and the goal-directed system, with maladaptive habits controlling behavior even in the face of aversive consequences^2,4^. However, recent experiments in rodents demonstrated that a response that had become habitual due to extensive training returned to a goal-directed action after a context change, implying that habits may be specific to the context in which they were acquired^97^. Hence, while we observed responding despite reward devaluation, indicating habitual behavior, we cannot rule out that a change of context would have reinstated goal-directed control. To further clarify the role of habits in gaming disorder, future studies may test the effect of outcome devaluation in a context different from the training context.

Finally, the use of oral contraceptives as well as the phase of the menstrual cycle have been found to not only affect the physiological response towards acute stress^76^ but also influence the effect of acute stress on cognition and emotion^33,98^. Future studies may explore whether these variables also modify the influence of stress on the PIT effect. Due to the small number of female participants in our sample, we were not able to address this question.

### Clinical implications

Our findings indicated that although the devaluation decreased reward-related responding, the PIT effect was not eliminated, i.e., the stimulus associated with gaming still increased responding for the gaming reward. This suggests that individuals who are motivated to change their problematic gaming behavior may encounter difficulties to actually do so. Up to date, one of the mostly studied treatments for gaming disorder is cognitive-behavioral therapy^99,100^. While there is support for its positive short-term effects on reducing symptoms of gaming disorder, there is initial evidence that the positive effects are no longer visible three to six months after treatment^101^. Habitual reactions towards gaming-related cues may be one mechanism that interferes with individuals’ attempts to effectively change their gaming behavior and these mechanisms may not be sufficiently addressed by cognitive-behavioral interventions. Thus, interventions are needed that target these mechanisms more directly.

Considering that approach-avoidance trainings have been effective in reducing approach biases towards gaming-related cues^102,103^, these interventions may also help to modify gaming-related PIT effects. However, two recent studies failed to find an effect of approach-avoidance trainings on the PIT effect, both in healthy participants^104^ and in patients with alcohol use disorder^105^. Given that the PIT effect is based on both instrumental and Pavlovian conditioning processes, it may be necessary to modify not only the instrumental behavior but also the stimulus-outcome associations. A promising intervention in this regard are emotional bias modification trainings, which have been shown to successfully decrease not only positive emotional association biases towards gaming cues but also compulsive gaming thoughts and behaviors^106^. Future studies may hence design and evaluate trainings that combine elements of approach avoidance and emotional bias modification in order to reduce the PIT effect.

Furthermore, our results emphasize the need for early intervention and prevention programs, as individuals with risky gaming behavior differed from individuals with unproblematic use, not only concerning gaming-related variables, but also with regard to impulsivity and psychological distress. Hence, even if only some individuals with risky gaming behavior may actually develop a gaming disorder^93^, early intervention programs can help to reduce risk factors, thus presenting an additional buffer against the development of gaming disorder.

## Conclusion

Our study found increased responding for gaming-related rewards in the presence of gaming-related cues in individuals with risky and non-problematic gaming behavior. In both groups, this gaming PIT effect was reduced, however, not eliminated after devaluation, which points towards a habitual character of the PIT effect. While stress did not promote habitual responding in participants aware of the stimulus-outcome associations, there was initial evidence for a stress effect on habitual behavior in unaware participants, which warrants future research.The persistent nature of the PIT effect may undermine potential attempts to change a problematic gaming behavior. Treatment of gaming disorder may hence benefit from the development of interventions or trainings that can reduce the PIT effect.

## Electronic supplementary material

Below is the link to the electronic supplementary material.

Supplementary Material 1

## Data Availability

Information where to find the dataset generated and analyzed in this study will be made available on https://osf.io/f27qw/?view_only=4bcea30152d54aab8d6c191e269cbe7d.
