# Supplementary material for: Transfer from goal-directed behavior to stimulus-response habits and its modulation by acute stress in individuals with risky gaming behavior
Source: Sci Rep. 2024 Oct 29;14:26015. doi: 10.1038/s41598-024-73899-3 (PMC11522379; doi:10.1038/s41598-024-73899-3)
Supplement: Supplementary file 1 — Supplementary Material 1 [file 41598_2024_73899_MOESM1_ESM.pdf]

Transfer from Goal-Directed Behavior to Stimulus-Response Habits and its Modulation by Acute Stress in Individuals with Risky Gaming Behavior

Anna M. Schmid, Tobias A. Thomas, Stefan Blümel, Nicolas K. Erdal, Silke M. Müller, Christian J. Merz, Oliver T. Wolf, Matthias Brand, Astrid Müller & Sabine Steins-Loeber

Supplemental Material

Results

Table S 1

Use of oral contraceptives and menstrual phase of female participants by group and condition

Variable	Groups			Conditions		
	Risky group (*n* = 9)	Control group (*n* = 10)	Group comparison	TSST (*n* = 7)	Placebo -TSST (*n* = 12)	Group comparison
Use of oral contraceptives, *n* (%)						
Yes	3 (33.3)	4 (40.0)	*p* = 1.00	4 (57.1)	3 (25.0)	*p* = .33
No	6 (66.7)	6 (60.0)		3 (42.9)	9 (75.0)	
Menstrual cycle phase[a], *n* (%)						
Follicular	0 (0.0)	2 (33.3)	*p* = .46	0 (0.0)	2 (22.2)	*p* = 1.00
Luteal	6 (100.0)	4 (66.7)		3 (100.0)	7 (77.8)	

Note. Due to the small sample size, Fisher's exact test as been used for group comparison. Two-tailed *p*-values are reported.

[a] The menstrual phase is only reported for female participants who indicated not to use oral contraceptives. The determination of the menstrual phase was based on forward count as described by Schmalenberger et al.[1], with day 1-16 of the menstrual cycle being classified as follicular phase, and day 17-28 being classified as luteal phase. One women who reported being on day 34 of her menstrual cycle was classified as being in the luteal phase.

Table S 2

Results of robust two-way mixed ANOVAs for stress response

Effect	Cortisol			Subjective stress		
	Q	*df*	*p*	*Q*	*df*	*p*
Condition	37.84	1, 56.95	<.001	8.51	1, 77.52	.005
Time	39.45	3, 50.73	<.001	44.42	3, 60.40	<.001
Time*Condition	27.64	3, 50.73	<.001	14.39	3, 60.40	<.001

Note. *n* = 132 for cortisol and *n* = 134 for subjective stress. Mixed ANOVAs on trimmed means had been computed using the bwtrim function of the WRS2 package in R.

Table S 3

Results of robust two-way mixed ANOVAs for response choice of the gaming-related reward in the instrumental training phase

Effect	*Q*	*df*	*p*
Group	8.16	1, 64.70	.006
Time	9.23	3, 64.84	<.001
Time*Group	0.24	3, 64.84	.87
Condition	0.06	1, 77.73	.80
Time	10.14	3, 67.04	<.001
Time*Condition	0.63	3, 67.04	.60

Note. *n* = 135. Mixed ANOVAs on trimmed means had been computed using the bwtrim function of the WRS2 package in R.

Table S 4

Response choice of the gaming-related reward before and after devaluation during the transfer phase

		Variable		Before devaluation		After devaluation		
Group	Condition	Awareness	Stimulus	*M*	*SD*	*M*	*SD*	*n*
Control	P-TSST	Unaware	Gaming stimulus	47.98	43.54	25.89	37.72	12
			Neutral stimuls	35.68	33.07	11.46	19.73	12
			Shopping stimulus	38.32	37.35	22.92	36.77	12
		Aware	Gaming stimulus	81.80	29.17	66.38	37.99	22
			Neutral stimuls	49.92	35.06	18.54	30.19	22
			Shopping stimulus	15.06	20.74	1.99	4.48	22
	TSST	Unaware	Gaming stimulus	42.79	52.31	38.75	48.09	5
			Neutral stimuls	56.75	38.24	3.92	5.92	5
			Shopping stimulus	25.00	39.28	16.00	35.78	5
		Aware	Gaming stimulus	77.19	35.93	55.82	42.78	27
			Neutral stimuls	51.23	38.17	17.67	26.98	27
			Shopping stimulus	10.41	22.58	3.86	13.11	27
Risky	P-TSST	Unaware	Gaming stimulus	60.69	29.75	33.84	32.61	9
			Neutral stimuls	68.68	22.28	35.69	28.10	9
			Shopping stimulus	59.17	31.72	39.58	33.51	9
		Aware	Gaming stimulus	88.50	23.16	76.02	34.76	25
			Neutral stimuls	53.28	31.08	14.72	18.77	25
			Shopping stimulus	15.03	27.62	9.30	20.73	25
	TSST	Unaware	Gaming stimulus	50.71	39.03	41.17	41.78	10
			Neutral stimuls	44.48	34.53	14.88	20.17	10
			Shopping stimulus	17.63	19.54	17.25	27.95	10
		Aware	Gaming stimulus	89.66	16.70	63.13	43.56	23
			Neutral stimuls	58.26	21.55	17.99	23.15	23
			Shopping stimulus	20.69	28.22	8.42	20.78	23

Note. Response choice of the gaming-related reward was calculated as percentage of trials in which the gaming-related response was chosen compared to all valid trials. Participants were classified as aware if they had learned which stimulus was associated with shopping and which stimulus was associated with gaming. P-TSST = Placebo Trier Social Stress Test. TSST = Trier Social Stress Test.

Table S 5

Results of robust two-way mixed ANOVAs for response choice of the gaming-related reward in the transfer phase

Effect	*Before devaluation*			*After devaluation*		
	Q	*df*	*p*	*Q*	*df*	*p*
Awareness	1.89	1, 25.58	.18	3.86	1, 30.27	.06
Stimulus	33.25	2, 24.82	<.001	29.03	2, 30.10	<.001
Awareness*Stimulus	10.19	2, 24.82	<.001	12.82	2, 30.10	<.001
Group	2.29	1, 52.90	.14	3.00	1, 77.55	.09
Stimulus	107.87	2, 61.05	<.001	42.56	2, 69.08	<.001
Group*Stimulus	0.06	2, 61.05	.94	0.43	2, 69.08	.65
Condition	0.12	1, 81.85	.73	0.67	1, 78.36	.41
Stimulus	150.68	2, 68.32	<.001	45.57	2, 71.70	<.001
Condition*Stimulus	1.44	2, 68.32	.24	0.03	2, 71.70	.97

Note. $n = 133$. Mixed ANOVAs on trimmed means had been computed using the bwtrim function of the WRS2 package in R.

1. Schmalenberger, K. M. *et al.* A systematic review and meta-analysis of within-person changes in cardiac vagal activity across the menstrual cycle: Implications for female health and future studies. *Journal of Clinical Medicine* **8**, 1946; https://doi.org/10.3390/jcm8111946 (2019).
